# Primary Sarcoma of the Appendix

**Published:** 1911-03-25

**Authors:** 


### Surgery.

### PRIMARY SARCOMA OF THE APPENDIX.

Malignant disease occurs in the appendix as in other parts of the alimentary tract, and similar to these parts it has been found that the vast proportion of cases are carcinomatous in nature. The subject was investigated by Harte in 1908, and at that time he was able to collect 111 cases of primary carcinoma of the appendix, whereas in the records he could find only six recorded cases of sarcoma, and some of these were doubtful. ("Annals of Surgery," vol. i. 1908). Summing up the evidence he came to the conclusion that, if carefully examined, from 0.3 to 1 per cent. of cases operated on for chronic appendicitis would be found to be malignant, or in other words carcinomatous, for it is stated that out of 7,000 appendix specimens submitted to pathological investigation at the Rochester Clinic, N.Y., not a single example of sarcoma occurred. The case recorded, therefore, by Dr. Powers in the *New York Medical Journal* for January 7, is not without interest. The patient was a girl, aged seventeen, who suffered, off and on, from sub-acute appendicitis for five weeks, only submitting to operation when persistent loss of weight (fifteen pounds in five weeks), and an acute exacerbation compelled surgical interference. The symptoms were general abdominal pain, associated with nausea and vomiting, and a moderately distended abdomen tender to the touch all over, the symptoms to which Sir Frederick Treves gave the name of "peritonism." No mass, or any special rigidity, was found in the right iliac fossa. The temperature was above normal, and a blood count showed leucocytosis to 14,000, with 86 per cent. of polymorphonuclear cells on a differential count. On opening the abdomen clear fluid was found in the peritoneal cavity, the intestines and omentum were ingested, and the picture suggested was that of commencing general peritonitis. The ordinary operation for removal was carried out, and apparently no glands were noticed at the operation. Microscopically the appendix was "very hard and large in the middle," with an "elastic nodular mass about the size of the tip of little finger, extending from the tip for 1¼ inch. Microscopic examination of the mass showed it to be a small round celled sarcoma, and this diagnosis was confirmed when the specimen was submitted to Professor Welch of the Johns Hopkins University. Examination of the free fluid found in the abdomen at the time of operation proved negative. The patient returned to her home two and a half weeks later, but steadily lost weight and strength. Gradually the abdomen became distended and hard, and a condition of general sarcomatosis was diagnosed. Systematic treatment with Coley's fluid was tried, but proved unavailing. She died ten weeks after the operation, or fifteen weeks after the first appearance of symptoms. Reviewing the other recorded cases it would seem that no age is exempt. It occurs in young children as well as those in the later decades of life. The growth, too, may vary. Round-celled, spindle-celled, and lymphosarcomatous growths have been found. Differential diagnosis appears to be difficult. All the cases seem to have presented the symptoms of sub-acute appendicitis. Persistent loss of weight and the presence of free fluid might cause one to be suspicious, but the diagnosis in most cases appears to have been made in the laboratory. As to the prognosis, it would seem to be very grave. One case was alive and well four years later, one case (Carwardine's) died nine months after of recurrence. A third was reported alive five months after operation; and the rest all died.

